# Can N-Methyl-D-Aspartate Receptor Hypofunction in Schizophrenia Be Localized to an Individual Cell Type?

**DOI:** 10.3389/fpsyt.2019.00835

**Published:** 2019-11-21

**Authors:** Alexei M. Bygrave, Kasyoka Kilonzo, Dimitri M. Kullmann, David M. Bannerman, Dennis Kätzel

**Affiliations:** ^1^Department of Neuroscience, Johns Hopkins University, Baltimore, MD, United States; ^2^Institute of Applied Physiology, Ulm University, Ulm, Germany; ^3^UCL Queen Square Institute of Neurology, University College London, London, United Kingdom; ^4^Department of Experimental Psychology, University of Oxford, Oxford, United Kingdom

**Keywords:** schizophrenia, psychosis, N-methyl-D-aspartate receptor, N-methyl-D-aspartate receptor hypofunction, parvalbumin, ketamine, MK-801, catatonic schizophrenia

## Abstract

Hypofunction of N-methyl-D-aspartate glutamate receptors (NMDARs), whether caused by endogenous factors like auto-antibodies or mutations, or by pharmacological or genetic manipulations, produces a wide variety of deficits which overlap with—but do not precisely match—the symptom spectrum of schizophrenia. In order to understand how NMDAR hypofunction leads to different components of the syndrome, it is necessary to take into account which neuronal subtypes are particularly affected by it in terms of detrimental functional alterations. We provide a comprehensive overview detailing findings in rodent models with cell type–specific knockout of NMDARs. Regarding inhibitory cortical cells, an emerging model suggests that NMDAR hypofunction in parvalbumin (PV) positive interneurons is a potential *risk factor* for this disease. PV interneurons display a selective vulnerability resulting from a combination of genetic, cellular, and environmental factors that produce pathological multi-level positive feedback loops. Central to this are two antioxidant mechanisms—NMDAR activity and perineuronal nets—which are themselves impaired by oxidative stress, amplifying disinhibition. However, NMDAR hypofunction in excitatory pyramidal cells also produces a range of schizophrenia-related deficits, in particular maladaptive learning and memory recall. Furthermore, NMDAR blockade in the thalamus disturbs thalamocortical communication, and NMDAR ablation in dopaminergic neurons may provoke over-generalization in associative learning, which could relate to the positive symptom domain. Therefore, NMDAR hypofunction can produce schizophrenia-related effects through an action on various different circuits and cell types.

## Introduction: The Glutamate Hypothesis of Schizophrenia

Schizophrenia is characterized by a wide range of symptoms, classically grouped into positive (hallucinations, delusions, disordered thought), negative (social withdrawal, anhedonia, apathy), and cognitive (deficits of attention, working memory, cognitive flexibility) domains (1, 2). Despite decades of research, the circuit basis of schizophrenia remains elusive, hampering progress in diagnosis and treatment.

No major breakthrough has been made in drug discovery for schizophrenia treatments since the introduction of the most effective atypical antipsychotic clozapine in 1971 (3, 4). All antipsychotic drugs currently approved for schizophrenia therapy have in common that they decrease signaling through dopamine D2 receptors (D2Rs), and are most effective on positive symptoms. However, negative and cognitive symptoms are largely resistant to currently available medication, and even positive symptoms respond only partially to the usual antipsychotic drugs, which in turn have significant side effects (1). Therefore, different treatment targets—not relying on dopamine receptor antagonism—are urgently required.

In that context, the so-called “glutamate hypothesis”—the assumption that aberrant glutamatergic signaling is at the core of schizophrenia pathology—has sparked the most hope. The only two new specific targets tested in phase III clinical trials in the last two decades in schizophrenia patients—the metabotropic glutamate receptor type 2/3 (mGluR2/3) (5–8) and the glycine transporter 1 (GlyT1) (9), which affects levels of the N-methyl-D-aspartate (NMDA) receptor (NMDAR) co-agonist glycine—result from work pointing to abnormal glutamatergic transmission in schizophrenia. A third target currently under investigation, D-amino acid oxidase (DAAO) (10), which also indirectly affects occupancy of the NMDAR co-agonist site, is also based on the “glutamate hypothesis.” A central pillar of this hypothesis is that hypofunction of NMDARs causes some or all of the symptoms of schizophrenia (11–13). NMDAR hypofunction may be a precursor to deficits in synaptic plasticity which have also been strongly linked to the disorder (14). The core question for preclinical research in this context is whether the NMDAR itself, or molecules that affect its activity, are appropriate drug targets in established schizophrenia. In turn, this can only be evaluated when the molecular phenomenon of NMDAR hypofunction is spelled out mechanistically in the context of neural circuits—i.e. when it is clarified which cell types in different brain structures are affected by NMDAR hypofunction and how such altered signaling could cause different aspects of the complex symptoms characterizing the disease.

## Linking NMDAR Hypofunction to Schizophrenia: Genetic Evidence

**Figure 1:**
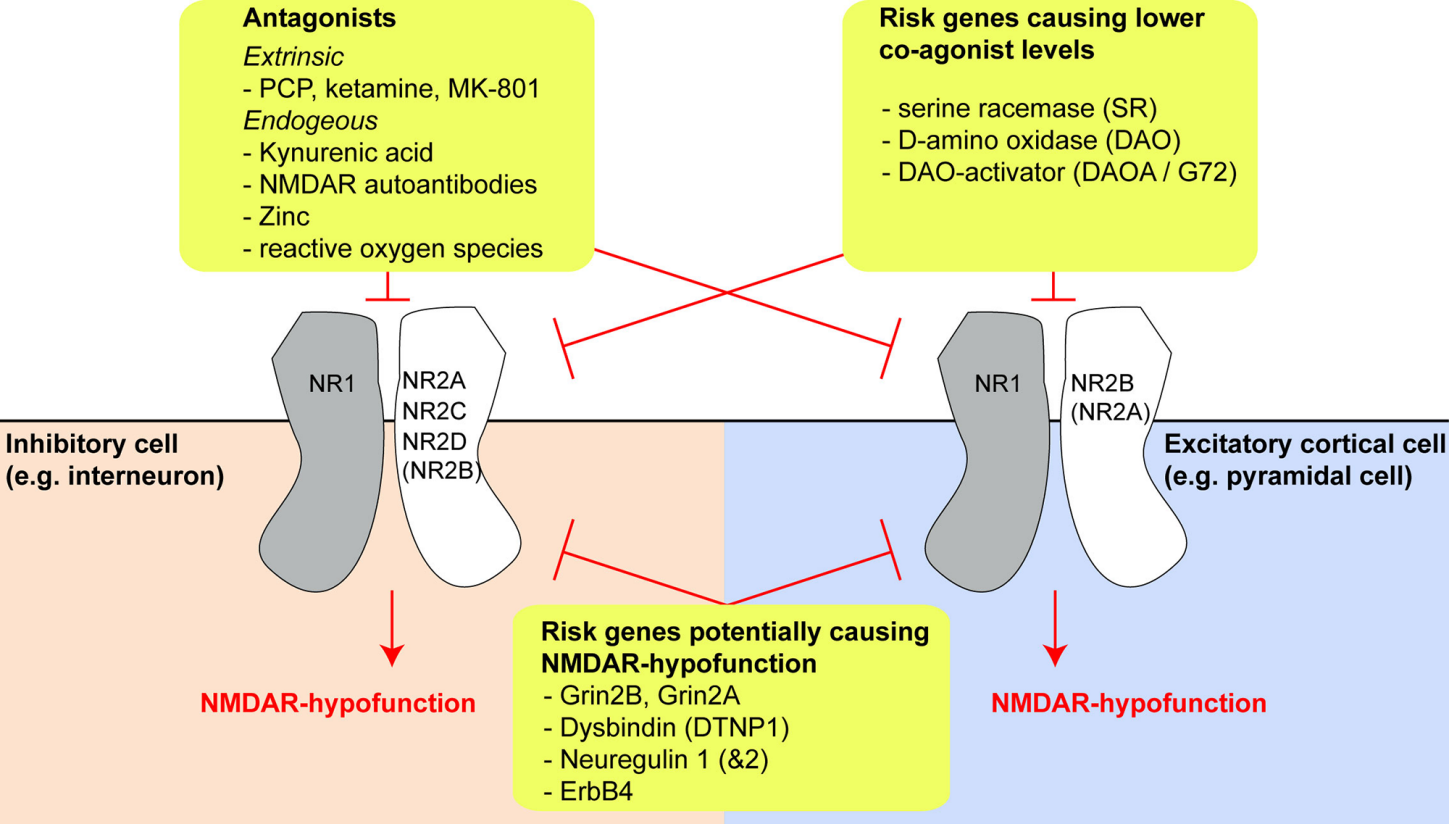
Risk genes and molecules producing N-methyl-D-aspartate receptor (NMDAR) hypofunction and their effects on different cell types. Overview of three different categories of risk genes or NMDAR-antagonistic molecules which may produce NMDAR hypofunction (yellow boxes). The expression of different GluN2-subunits by different cell types may be a key driver of differential vulnerability of specific types of neurons in schizophrenia. The extent of GluN2B expression in interneurons and GluN2A expression in pyramidal cells is uncertain, as conflicting results exist in the literature and public databases, but they are likely to be small, at least in prefrontal cortex (see main text).

The glutamatergic synapse, and in particular the NMDAR, is one of the most prominent points of convergence of genetic risk factors for schizophrenia (15–17). Reduced expression of the obligatory NMDAR subunit GluN1 (also known as NR1; encoded by *GRIN1*) and also of GluN2C (NR2C, encoded by *GRIN2C*) has been reported in post-mortem prefrontal cortex tissue of schizophrenia patients (18, 19). In addition, the *GRIN2A* and *GRIN2B* genes, encoding the GluN2A (NR2A) and GluN2B (NR2B) subunit of the NMDAR respectively, are identified schizophrenia risk genes (**Figure 1**) (16, 17).

At least one *GRIN2B* risk allele (rs1805502) is associated with decreased expression of GluN1 in patients (18). Moreover, exome-sequencing studies revealed that rare damaging mutations in *GRIN1*, *GRIN2A*, and *GRIN2B*, which are expected to cause NMDAR hypofunction directly, are found in samples from schizophrenia patients, but not controls (20, 21).

Experiments in mice provide further support for a link between schizophrenia and hypofunction of GluN2A and GluN2B: Global ablation of *Grin2A* causes spatial working/short-term memory deficits (without impairing basic spatial processing) (22). Further, pharmacological blockade of GluN2B alone is sufficient to cause behavioral abnormalities in rodents, in particular increased locomotor activity, which has been likened to psychosis (23, 24), as well as reduced cognitive flexibility (25), but does not recapitulate the entire profile of schizophrenia, at least as far as the disease can be modeled in rodents. For instance, pharmacological GluN2B blockade does not affect pre-pulse inhibition and may increase motivation, working memory, processing speed, sustained attention, and also motor impulsivity (23, 24, 26–29)—note, however, that these pharmacological results can contrast strongly with the schizophrenia-related deficits seen with cell type–specific *Grin2B*-ablation in mice (see below).

Risk genes other than those encoding NMDAR subunits may affect the production or degradation of D-amino acids that act as obligatory co-agonists at the NMDAR, especially of D-serine (**Figure 1**). This includes the genes encoding the D-serine producing enzyme *serine racemase (SR)*, the D-serine degrading enzyme *D-amino acid oxidase* (DAAO, encoded by *DAO*), and its activator DAOA, also termed G72 (30). Four other risk genes—dysbindin (*DTNP1*), neuregulin 1 (*NRG1*), neuregulin 2 (*NRG2*), and the NRG1/2-receptor ErbB4 (*ERBB4*)—are implicated in regulating NMDAR function and synaptic plasticity, and their expression is altered in post-mortem tissue in schizophrenia: downregulation of *DTNP1* in hippocampal and cortical tissue has been reported in schizophrenia patients, and *Dtnp1* knockout in mice leads to reduced expression of NMDARs and correlates with working memory impairments (31, 32). Increased neuregulin 1–ErbB4 signaling has been reported in the frontal cortex in patients and suppresses NMDAR activation (33, 34), modulating GluN2B subunits in particular (35). Two studies also found increased neuregulin 1 expression in post-mortem tissue from prefrontal cortex (36) and hippocampus (37) of schizophrenia patients, although a third did not detect this pattern (35).

Similarly, neuregulin 2 expression leads to internalization of GluN2B-containing NMDARs in cortical interneurons, but is also itself decreased by NMDAR activation (38). Neuregulin 2 expression is, however, *reduced* in schizophrenia patients (36), and *Nrg2* knockout in mice leads to a wide range of schizophrenia-related and partly clozapine-responsive deficits (reduced prefrontal dopamine, novelty- and amphetamine-induced hyperlocomotion, and impairments of T-maze working memory, sociability, and pre-pulse inhibition) (39). Finally, exome sequencing has revealed rare schizophrenia-related damaging mutations in the gene encoding the tyrosine-kinase *FYN*, which regulates NMDAR-trafficking (20, 21).

## Linking NMDAR Hypofunction to Schizophrenia: Pharmacological Evidence

Support for the NMDAR hypofunction hypothesis of schizophrenia also comes from the observation that ketamine and phencyclidine (PCP), which are use-dependent non-competitive blockers of the NMDAR-channel, induce cognitive, negative, and positive symptoms of the disease (and increased dopamine signaling) in humans and rodents (13). The finding that dopaminergic agonists, in contrast, may only reproduce *positive* symptoms is central to the proposal that NMDAR hypofunction is causally upstream of dopaminergic aberrations in schizophrenia (11, 40, 41). Importantly though, both PCP and ketamine have also been reported to act as agonists on D2Rs as well, they display a similar affinity for D2Rs as for NMDARs and a considerably higher affinity for the high dopamine-affinity functional state D2R^High^ (42–45). Such D2R-agonism could contribute synergistically to the psychotomimetic effects of PCP and ketamine. Also, low-affinity binding of both drugs to 5-HT2 receptors has been suggested (42) but has not been confirmed by others (46). Low-affinity binding of PCP to the serotonin transporter (SERT) has also been found (47, 48), but the contribution to its psychotomimetic effects are unclear. Finally, ketamine—through its main metabolite hydroxynorketamine—also increases expression of prefrontal and hippocampal AMPA glutamate receptor subunits, alters striatal BDNF signaling, and—just like ketamine itself—increases G_s_-protein mediated signaling, including cAMP response element binding (CREB) protein levels, independently from NMDARs (49, 50).

The similarity between the psychological effects of NMDAR blockers in humans and symptoms of schizophrenia was described in a large review of over two dozen studies, which report the effects of PCP when used as an anesthetic or recreationally, or when administered experimentally in schizophrenia patients (51). This evidence was complemented with several subsequent controlled studies using ketamine (11, 52–54). Some studies showed that ketamine (55) or PCP (51, 56–58) may re-institute psychotic symptoms in therapeutically stabilized patients with schizophrenia. Notably, in healthy humans, ketamine induces a resting state connectivity pattern (especially involving the prefrontal cortex) that resembles the brain state of the prodrome and early-stage schizophrenia, rather than the chronic brain state in schizophrenia (59).

Furthermore, there is evidence that maternal PCP abuse during pregnancy (putatively causing NMDAR hypofunction in the developing brain) increases the risk for developing schizophrenia later in life (60). Complementary rodent studies show that NMDARs have a crucial role in virtually *all* processes of cellular neuronal development (61, 62), leading to alterations of neuronal circuits (63) and behavior later in life (64).

## Schizophrenia Does Not Equal NMDAR Blockade: a Need to Refine the Framework

While the evidence summarized above supports an association between NMDAR hypofunction and schizophrenia, several recent studies call for qualification of some earlier conclusions.

*Firstly*, the *specific* deficits seen with PCP and ketamine do not *precisely* recapitulate the specific symptoms seen in patients with schizophrenia. The apparent discrepancy might, at least in part, result from over-generalization during symptom classification, for instance when referring too airily to “symptom domains.” One recent study evaluated the experiences reliably induced by ketamine—as reported by recreational drug users; the experience items of the questionnaire used in this study had previously been matched to distinct psychiatric disorders by professional psychiatrists. Notably, ketamine was not reported to reliably induce experiences that cluster specifically within any of the cognitive, positive, or negative domains of schizophrenia as opposed to other psychiatric disorders (e.g. depression) (65). In fact, non-glutamatergic drugs such as psilocybin and amphetamine provided a better match (65).

Another study demonstrated that the psychotic experiences induced by ketamine, especially body image disturbances and “out-of-body” experiences—also described in previous PCP-studies (51, 66)—do not correspond to psychotic symptoms seen in schizophrenia (66). The effects seen after higher doses of PCP (e.g. 10 mg PCP i.v.) may be better categorized as “delirium” as defined by *DSM-V* (66, 67). There is also evidence that the use of ketamine as an anaesthetic seems to be tolerated by schizophrenic patients at least as well as, if not better than, depressed subjects (68). The *specificity* of symptom-matching required to evaluate the suitability of this pharmacological model also raises a note of caution for *preclinical* research, which does not even come close to the level of precision in measuring psychological deficits achieved in humans.

*Secondly*, there are questions surrounding data from mouse models designed to exhibit reduced NMDAR function. So-called NMDAR hypomorph mice which have a 90–95% reduction of NMDAR levels exhibit a very wide range of behavioral and physiological abnormalities not restricted to those related to schizophrenia (69–74). Indeed, there were no behavioral assays on which these mice performed normally. Thus, these mice exhibit a global behavioral impairment, and it is impossible to determine what psychological processes are disrupted in these mice. For instance, they also show altered ultrasonic vocalizations and increased repetitive behavior which are more suggestive of autism (75). Likewise, electrophysiological biomarkers of both schizophrenia and autism are found (69, 75). In essence, these mice may not show a *specific* schizophrenia-related endophenotypic profile.

*Thirdly*, although the identification of *anti-NMDAR encephalitis* as an important neurological disease with marked psychotic features (76, 77) at first sight provides a direct link to schizophrenia (78), the syndromes exhibit important differences. These patients develop an autoimmune disorder associated with antibodies targeting the extracellular domain of the obligatory NR1 (GluN1) subunit present in all NMDARs, leading to receptor clustering and internalization (79). In primary neuronal cultures, patient antibodies can reduce the surface expression of NR1 in both excitatory and inhibitory neurons (80). Some patients experience symptom patterns that are remarkably similar to schizophrenia—including positive symptoms such as hallucinations, delusions, and agitation; deficits in attention, executive functioning, working, and short-term memory present in around 50% of patients (77, 81–83). A further common endophenotype of both diseases is increased delta oscillations (84, 85). However, these schizophrenia-like symptoms do not occur in *all* patients with anti-NMDAR encephalitis, and negative symptoms are rather rare (86). Furthermore, deficits unrelated to schizophrenia such as hyper-religiosity, strong speech reduction and disintegration, high anxiety and impulsivity, epileptic seizures, breathing problems, and autonomic instability are common. Given the frequent occurrence of rigidity, mutism, or dystonia (83), there might be parallels to catatonic schizophrenia (a sub-category, which has, however, been separated from schizophrenia in *DSM-V*), but, overall, NMDAR encephalitis appears to extend beyond and is not a perfect match for schizophrenia (86). This conclusion is supported by the very different temporal trajectories of pathologies in the two disorders with a rapid onset of symptoms in NMDAR encephalitis (86) but a more prolonged (likely neurodevelopmental) disease course over multiple years in schizophrenia (87).

A *final* note of caution derives from the limited clinical success of pharmacological enhancement of NMDAR function. Several phase III clinical trials evaluating the GlyT1 inhibitor bitopertin, which elevates extrasynaptic levels of the NMDAR co-agonist glycine, have failed to show significant symptom improvements (9, 88). Moreover, a meta-analysis of the therapeutic efficacy of glycine, D-serine, and other NMDAR co-agonists or positive modulators revealed that—while these drugs had small-to-medium significant beneficial effects when given to non-clozapine-treated schizophrenia patients (with glycine having qualitatively a larger effect-size than D-serine)—none of the approaches was superior to therapy with clozapine (89); for a review, see (90). In line with these results, knockout mice lacking serine racemase failed to show key deficits expected for a mouse model of schizophrenia, including no impairment of social interaction or various forms of short-term memory (91). Furthermore, the evidence that the glycine-binding site can be activated by these drugs is actually quite weak. Indeed, the lack of strong effects might be due to the fact that this binding site is already largely saturated (92, 93). On the other hand, the DAAO inhibitor sodium benzoate, which blocks degradation of D-serine, has shown promising effects on positive and negative symptoms (10). Several clinical trials with DAAO inhibitors are underway. Providing a potential proof of concept for DAAO inhibitors, genetic ablation of DAAO in mice results in enhancement of multiple forms of short-term memory—but not long-term memory (94, 95).

In summary, numerous studies link NMDAR hypofunction to schizophrenia-related deficits in humans and animal models, but the actual nature and extent of its contribution to the causation of human schizophrenia remains uncertain. The effects of global NMDAR antagonism *exceed* the symptom spectrum of schizophrenia. This discrepancy might be explained if NMDAR hypofunction in human schizophrenia is only partial (i.e. weaker than, for example, in anti-NMDAR encephalitis), and potentially causes aberrations across many years of learning and brain development, reflective of the neurodevelopmental nature of schizophrenia (87). Another possibility explored in the remainder of this review is that schizophrenia might be associated with NMDAR hypofunction in *only some* cell types of the brain, but not others. These alternative explanations are, of course, not mutually exclusive.

## Does NMDAR Blockade Exert Its Effects Primarily Through Interneurons?

**Figure 2:**
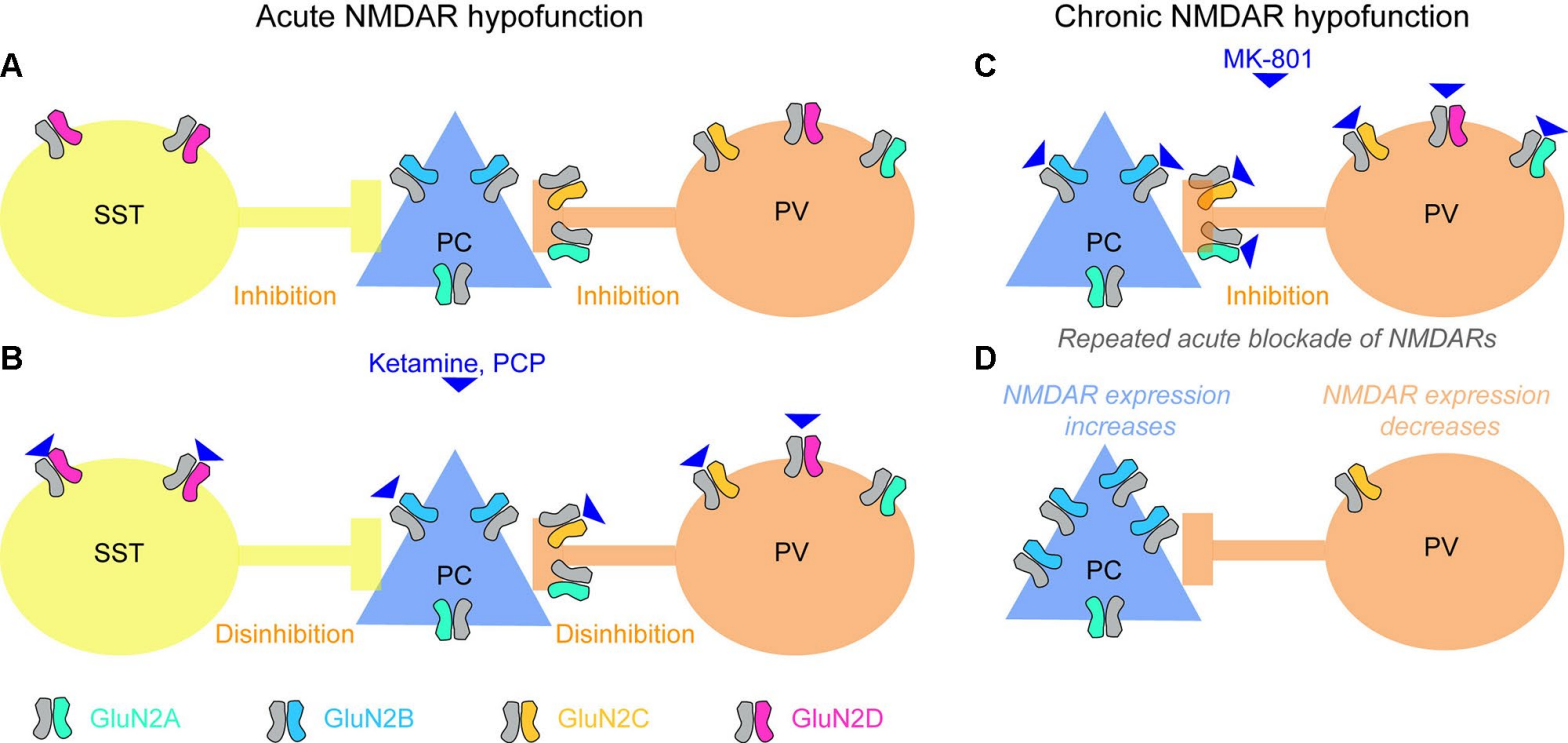
Effects of acute and chronic blockade of NMDARs. **(A)** Baseline state with GluN2 NMDA receptor subunit types most prevalent in each of the three cortical neuron classes (somatostatin-positive interneuron, SST; glutamatergic pyramidal cells, PCs; parvalbumin-positive interneuron, PV) color-coded. SST and PV interneurons provide inhibition to pyramidal cells. **(B)** PCs express mainly GluN2B-containing NMDARs that are less sensitive to ketamine and strongly blocked by magnesium ions at normal (hyperpolarized) resting membrane potential; in some areas they might also express GluN2A subunits, which are similar in terms of sensitivity to magnesium and ketamine. NMDARs containing GluN2C (on PV cells) and GluN2D (on PV and SST cells) (78, 102–106), in contrast, may be more sensitive to ketamine (107), and are hardly blocked by magnesium (107–109), therefore contributing to the glutamate-mediated excitation of these cells even at non-depolarized resting potential. [Although note that a recent RNAseq study questions the notion that GluN2C/D are strongly expressed in neocortical PV cells (110, 111)]. Consequently, when ketamine is applied, glutamatergic excitation of SST and PV cells could be strongly reduced, while excitation of PCs is not altered much, and the activity in the circuit increases due to disinhibition. Note, that—as an important qualification of this model—NMDARs are only weakly expressed by the somata of PV cells, but they occur in their presynaptic terminals, and their blockade reduces release of their inhibitory neurotransmitter GABA (see main text). Also, this model is complicated by the fact that the affinities of different GluN2-subunits differ for the two NMDAR blockers frequently used to model schizophrenia, ketamine and MK-801, and, furthermore, that the relevant literature is inconsistent. While one study reported nearly equal efficacies of MK-801 for all GluN2-subunits (112), another study reported nearly equal efficacies for ketamine but a 10-fold higher efficacy of MK-801 on GluN2A/B relative to GluN2C/D (108, 113). A more recent study demonstrated a high selectivity of ketamine for GluN2C/D over GluN2A/B, and additionally reported that the discrepancy with the earlier study might have been due to lack of Mg^2+^ (114, 107). Also note that a further blocker applied frequently in both humans and animals, phencyclidine (PCP), seems to cause schizophrenia-related abnormalities preferentially by blockade of GluN2D-NMDARs (115), similarly to ketamine (116). **(C**, **D)** Repeated blockade of NMDARs on pyramidal cells and PV interneurons **(C)** results in an adaptive *increase* of NMDAR expression in PCs, but a *reduction* of NMDAR expression in PV cells **(D)** (117). This means that the contribution that NMDARs make to the excitation of these two neuron classes shifts, which could be responsible for the observed overactivity of the hippocampus produced by chronic ketamine, and could make the circuit prone to different responses (e.g. leading to catatonic states of schizophrenia) when global blockade of NMDARs occurs again (see main text). Note, however, that the adaptation shown in **(C**, **D)** has been shown in prefrontal cortex using MK-801, while the mechanisms in acute blockade **(A**, **B)** largely reflect data collected in the hippocampus and usage of ketamine or PCP.

Pharmacological blockade of NMDARs by ketamine or PCP leads to an increase in extracellular glutamate levels (96). Elevated glutamate in the hippocampus and prefrontal cortex has also been suggested to occur in patients with schizophrenia, measured using magnetic resonance spectroscopy (MRS) (97), although the relationship between glutamate detected using MRS and extracellular (synaptic or extrasynaptic) levels of glutamate remains uncertain. In rodents, NMDAR blockade has been reported to lead to increased activity of excitatory neurons and release of glutamate (98–101). This counterintuitive finding, that the blockade of a major excitatory ionotropic receptor leads to an *elevated* activity of cortical excitatory cells releasing glutamate, has been explained by a preferential decrease of NMDAR-mediated excitatory drive on to *inhibitory* cells within the circuit, leading to a net disinhibition of excitatory cells (78, 99) (**Figure 2**).

So far, however, this *local* disinhibitory mechanism has only been demonstrated in the CA1-network of the hippocampus (101). The observed increase of excitatory activity in prefrontal cortex (mPFC) (99) appears to be a consequence of NMDAR blockade in the hippocampus or/and thalamus, rather than local disinhibition in the mPFC (101, 118–121).

Both acute and chronic application of ketamine in mice increases glutamate release in the ventral hippocampus, corresponding to hyperactivity of the anterior hippocampus in patients (98). However, while *acute* blockade of NMDARs leads to elevated dopamine release in the prefrontal cortex (122, 123), *repeated* treatment blunts prefrontal dopamine release and—in contrast to *acute* application—also decreases mismatch-negativity and both the power and coherence of gamma oscillations (124). Given that schizophrenia patients also show *reduced* prefrontal dopamine release (125), the *adaptation to* NMDAR hypofunction, rather than—or in addition to—NMDAR hypofunction itself, could be important to the pathophysiology of the disease.

## Localizing Schizophrenia-Related NMDAR Hypofunction to Parvalbumin-Positive Interneurons

To date the most influential version of the NMDAR hypofunction hypothesis of schizophrenia localizes this defect to interneurons that express the calcium-buffer protein parvalbumin (PV, encoded by *PVALB*), which are typically fast-spiking and implicated in gamma oscillations (41). PV interneurons appear to react uniquely when NMDAR hypofunction is induced globally: For example, in rodents PV expression is reduced following MK-801 (126–128), PCP (129), or ketamine treatment (130, 131) [although see (132)], potentially due to hypermethylation of the *Pvalb* promoter (133). This recapitulates the reduced expression of PV found *post-mortem* in cortical tissue from schizophrenia patients (134, 135), which is expected to alter GABAergic output from PV interneurons given that PV buffers calcium during the high-frequency firing of these interneurons (41, 136, 137). Also, acute NMDAR antagonism by MK-801 leads to a chronic reduction of NMDAR expression in PV interneurons but not in the vast majority of other neurons (117). Furthermore, increased expression of NRG1 in transgenic mice, reproducing an endophenotype in patients (36, 37), leads to reduced NMDAR expression in PV-positive basket cells of the hippocampus, although CCK-positive cells also show this pattern (138). Interestingly, the NRG1-receptor ErbB4, encoded by a schizophrenia risk gene (16), is preferentially expressed in PV and other hippocampal interneurons, but not pyramidal cells (139), and may regulate NMDAR expression also in response to the NRG1-homolog NRG2, which is expressed by those interneurons themselves (38).

## From NMDAR Hypofunction to PV Interneuron Hypofunction

**Figure 3:**
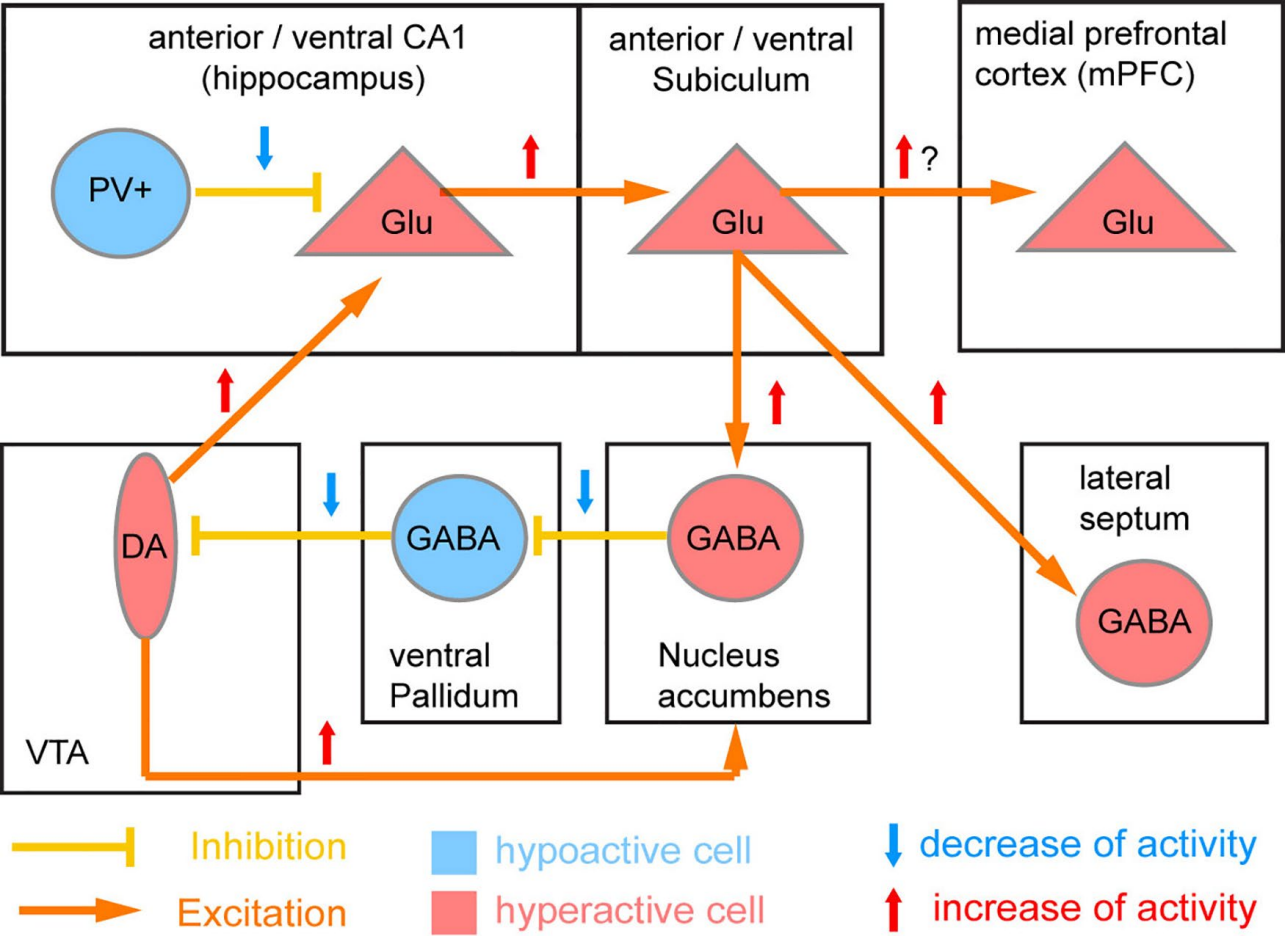
Circuit model of NMDAR hypofunction induced deficit of PV neurons. The original model by Lisman et al. (41) proposed that parvalbumin-positive (PV) interneurons of the CA1 region of the human anterior (rodent ventral) hippocampus is the primary location of NMDAR hypofunction in schizophrenia. Reduced NMDAR activity in these PV cells is supposed to lead to a downregulation of PV and Gad67 expression and thereby of the GABAergic output of PV cells and resulting disinhibition of surrounding excitatory glutamatergic (Glu) cells. The resulting disinhibition of CA1 excitatory glutamatergic projection neurons would lead to overactivation of the output stage of the hippocampus (subiculum) as seen in patients (98), and through a disynaptic loop through the basal ganglia cause a hyperactivity of ventral tegmental area (VTA) dopamine (DA) neurons innervating the nucleus accumbens (potentially causing psychosis) and also the hippocampus (positive feedback loop).

The PV cell population itself includes several subtypes, and there is continuing uncertainty regarding which population and which brain region (the hippocampus, the prefrontal cortex, the neocortex as a whole, or other brain areas such as the thalamus) is the most relevant. The most prominent account (41), which has become known as the “Lisman/Grace model,” assumes that NMDAR hypofunction in PV interneurons of the CA1-subfield of the human anterior hippocampus (corresponding to the rodent *ventral* hippocampus) is the key starting point of schizophrenia pathology (**Figure 3**).

From the outset, this seems to be an unlikely scenario, since PV cells have very low expression of NMDA receptors compared to other neuron types in this region (140). In fact, PV interneurons across the brain (in common with other medial ganglionic eminence–derived interneurons) have relatively small NMDAR-mediated currents and, conversely, a high synaptic calcium current through inward-rectifying calcium-permeable AMPA-receptors (141). This implies that a reduction of NMDAR expression might not be expected to reduce the excitation or calcium influx in PV cells appreciably. However, it is important to note that there are input-specific differences in the magnitude of NMDAR currents recorded in CA1 PV interneurons (142). More specifically, Schaffer collateral feed-forward inputs onto PV-basket cells—as recorded in (141)—have vastly lower NMDAR currents compared to feedback inputs from within CA1 (142, 143). This likely has consequences for dendritic computations in PV interneurons, as has been shown with NMDAR-dependent supra-linear summation of local CA1 inputs (143).

However, a key twist in this model (which posits a deficit of NMDAR signaling in PV cells as a core pathological mechanism, **Figure 3**) is the assumption that NMDARs in such PV interneurons serve primarily as *sensors* of the overall glutamatergic activity present in a local circuit—thereby obviating the need for a receptor to be present in large quantities in order to have strong effects (41). Lower activation of NMDARs and a resulting decrease in calcium entry could signal reduced overall *excitatory* network activity and lead to a homeostatic downregulation of *inhibitory* output from PV cells in order to maintain the excitation/inhibition balance. Reduced expression of the primary GABA-producing enzyme GAD67 (encoded by *GAD1*) and PV could then reflect adaptive reductions of inhibitory output, and are indeed seen in *post-mortem* cortical tissue from schizophrenia patients (134, 144, 145). While the number of neocortical PV interneurons does not seem to decrease in schizophrenia, about half of them lack GAD67 (at least in prefrontal and anterior cingulate cortex) (135, 146, 147), and may thereby lose their ability to exert an inhibitory influence on the network (148, 149). Notably, the expression of GAD67 and PV seems to be constantly changing even under physiological conditions, depending on sensory experience and memory formation (150). This implies that a pathological situation that decouples NMDAR activity in PV cells from actual glutamate release in the circuit could lead to a maladaptive reduction of GABA release from PV interneurons (41).

The nature of this decoupling is unclear. Post-mortem findings in schizophrenia have reported reduced NMDAR expression in multiple cell types, including interneurons, but notably PV cells were not disproportionately affected (146, 151, 152). However, the GluN2C subunit, which is much more prominently expressed in cortical PV interneurons than pyramidal cells according to some studies (102, 103, 117)—but not others (110, 111)—is indeed downregulated in the prefrontal cortex (18). Alternatively, a decrease in levels of NMDAR co-agonists (D-serine, glycine; for example due to genetic changes in the schizophrenia risk genes *SR*, *DAO*, or *DAOA/G72*) or increased levels of endogenous NMDAR blockers such as zinc or kynurenic acid, could play a role (**Figure 1**). The tryptophan-degradation metabolite kynurenic acid, which is also an endogenous non-specific antagonist of the NMDAR glycine site, exhibits elevated levels in the cortex (153) and cerebrospinal fluid (154) in patients with schizophrenia. Application of kynurenic acid induces spatial working memory deficits in rodents (155), while blockade of its production enhances cognition (156) and reduces firing activity of ventral tegmental area (VTA) dopamine neurons, as would be predicted from a specific augmenting action on inhibitory neurons of the ventral hippocampus (41, 157). However, it is unclear why such global NMDAR hypofunction should affect PV cells with relative specificity.

In the Lisman/Grace model (**Figure 3**), at the circuit level the proposed key downstream consequences of the resulting disinhibition of CA1 pyramidal cells are hyperactivity of the anterior hippocampal output stage, the subiculum, and excess activation of dopamine neurons in the VTA resulting from a disinhibitory loop through the nucleus accumbens and the ventral pallidum (41). Virtually all of those physiological aberrations have been documented in patients with schizophrenia, and the control of VTA dopamine neurons by the subiculum has been established in rodents (158, 159). However, it is as yet unclear if such a hyperactivity of the hippocampal output actually results from a decrease of inhibitory output from CA1 PV interneurons. While direct chemogenetic silencing of hippocampal PV interneurons failed to evoke behavioral correlates of elevated VTA-dopamine activity—elevated locomotion and amplification of amphetamine-induced locomotion—inhibition of *other* hippocampal interneurons (specified by *GAD2*-expression) was able to do so (160). Also, putative blockade of GluN2A-containing NMDARs, which are particularly prominent in PV interneurons (117, 161), does not appear to cause hyperlocomotion at all (23). On the other hand, mouse models in which hippocampal PV interneurons are permanently reduced in numbers *did* recapitulate core deficits of schizophrenia, including hippocampal hyperactivity and increased novelty-induced hyperlocomotion which was partially resistant to anti-dopaminergic treatment (162, 163). It should be noted that although the characteristic increase of locomotion (a rodent correlate of positive symptoms) seen with stimulation of the ventral subiculum is usually attributed to increased VTA dopaminergic activity (41, 159), the locomotion-inducing effect of NMDAR blockers may not entirely require intact dopamine release given that it is also apparent in dopamine-depleted rodents (164–167).

Other versions of this hypothesis propose that NMDAR hypofunction is instead localized to *prefrontal* PV interneurons (134, 145, 168–170). Decreased PV expression has so far been documented in *neocortical* tissue from schizophrenia patients rather than in the hippocampus (146–148). It has been suggested that weakening of the output of prefrontal PV interneurons would lead to less synchronized output from prefrontal projection neurons targeting a specific subset of dopamine and GABAergic neurons in the VTA (168). Given their known target specificity, this would be expected to result in reduced activation of prefrontal-projecting (mesocortical) dopaminergic neurons and concomitant disinhibitory activation of (mesolimbic) dopamine cells projecting to the nucleus accumbens, thereby giving rise to the well-documented co-existence of hypofrontality and striatal hyperdopaminergia in schizophrenia (168). However, a direct demonstration of this mechanism is so far lacking. Related accounts focused rather on the link between NMDARs in PV interneurons and gamma oscillations, which are relevant to various cognitive functions and are disrupted in schizophrenia (145, 169–171).

Finally, a recent study showed that NMDARs are expressed *presynaptically* by prefrontal PV interneurons innervating pyramidal cells, and that their activation can enhance evoked GABA release (i.e. PV cell–mediated inhibition), and their blockade by MK-801 reduced inhibitory currents in pyramidal cells, if (and only if) glutamate was released by surrounding neurons (172). This finding could resolve the long-standing puzzle as to how *acute* application of NMDAR antagonists could lead to an effective PV disinhibition of pyramidal cells, despite the fact that NMDARs might not contribute significantly to the somatodendritic excitation of PV interneurons.

## Genetic Modeling of NMDAR Hypofunction in Cortical Interneurons

In order to assess whether NMDAR hypofunction in PV-positive interneurons—during both postnatal development and adulthood—can indeed cause core aberrations of schizophrenia, several laboratories have now deleted the obligatory NMDAR subunit GluN1 (and thereby all NMDARs) selectively from PV-positive interneurons in mice. The principal method to achieve this is to cross a mouse line in which an exon of the GluN1-encoding gene *Grin1* is floxed, to a mouse line that expresses *Cre*-recombinase selectively in PV-positive interneurons. In the resulting double-transgenic mice, NMDARs are ablated only in neurons where *Cre* is expressed. The time at which this occurs depends on when the promoter that drives *Cre* is activated and on the location of the lox-sites within the *Grin1* gene because recombination is a stochastic event whose probability increases the closer the lox-sites are to each other (78, 173). Because the average lifetime of an individual NMDAR-molecule in the post-synaptic membrane is not known, it is difficult to estimate precisely the actual time course of the removal of those NMDARs that had been produced before the gene disruption. In practice, strategies to delete NMDARs from PV interneurons have placed *Cre* under the control of the promoter of either the *Ppp1r2* gene, with expression onset at postnatal day (P) 7, or the PV gene (*Pvalb*) with an estimated onset between P10 and P14 in neocortex (174), generating *Grin1^ΔPpp1r2^* or *Grin1^ΔPV^* mice, respectively. In our experiments using the *Pvalb* promoter, a significantly reduced NMDAR-mediated current in PV cells could be measured at 2 months of age, although some PV interneurons did still show NMDAR-mediated currents, despite the comprehensive coverage of the PV cell population by the genetic driver line that was used (175).

## PV-Specific NMDAR Ablation Alone Does Not Reliably Induce a Schizophrenia-Related Spectrum of Deficits

Surprisingly, selective genetic deletion of NMDARs in PV interneurons in mice has, by and large, *not* supported the model that NMDAR hypofunction in these cells is *sufficient* to mimic schizophrenia. **Table 1** summarizes the results of the behavioral assessment of these mouse lines published to date. In our hands, using either the *Ppp1r2*-Cre (176) or the more commonly used *Pvalb*-Cre (175) line to drive GluN1 ablation, there were hardly any behavioral deficits across a wide spectrum of rodent correlates of positive, negative, and cognitive symptoms when the mice were raised in enriched environments (that is, involving a shelter, such as a house and/or tube, and nesting material). This is largely supported by the results from three other labs that have used the same *Pvalb*-Cre mouse line, albeit in combination with a floxed-GluN1 line that featured a larger distance between lox-sites (171, 177–179); see **Table 1**. A possible exception is a deficit in alternation-based spatial working memory assays, which has been seen in either strong (180) or very subtle (171, 175, 176) forms by some labs but not by others (177, 178).

**Table 1:** Deficits induced by putative genetic N-methyl-D-aspartate receptor (NMDAR) ablation in parvalbumin interneurons. Results of behavioral tests conducted in the indicated studies (top row) measuring rodent correlates of schizophrenia in the positive, cognitive, and negative domain as well as anxiety (see left two columns) in distinct double-transgenic conditional knockout lines (stated in rows 2–4). Green →, no change; magenta, schizophrenia-related deficit; orange, deficit provoked or exacerbated by environmental stress; blue, apparent improvement of function or opposite of the expected. ↑, increase of behavioral measure; ↓, decrease of behavioral measure. *Studies*: Belforte 2010 (173), Billingslea 2014 (178), Bygrave 2016 (175), Bygrave 2019 (176), Carlen 2012 (171), Jiang 2013 (181), Korotkova 2010 (180), Saunders 2013 (177), Pozzi 2014 (179). *Cre-Driver lines*: Ppp1r2 (Jax# 012686) (173), PV (Monyer) (182, 183), PV (Arber, Jax# 008069) (175, 184, 185). *Floxed-Grin1 responder lines*: Seeburg (186), Li (187), Tonegawa (Jax# 005246) (188).

	Publication	Belforte 2010 Jiang 2013	Belforte 2010	Bygrave 2019	Korotkova 2010	Carlen 2012	Saunders 2013	Billingslea 2014	Pozzi 2014	Bygrave 2016
Line	Driver	Ppp1r2	Ppp1r2	Ppp1r2	PV (Monyer)	PV (Arber)	PV (Arber)	PV (Arber)	PV (Arber)	PV (Arber)
	Responder	Li	Tonegawa	Seeburg	Seeburg	Tonegawa	Tonegawa	Tonegawa	Tonegawa	Seeburg
	loxP-to-loxP distance	2.1 kb	12 kb	3.3 kb	3.3 kb	12 kb	12 kb	12 kb	12 kb	3.3 kb
Positive	Novelty-induced LMA	↑ periphery; ↓center*	–	→	→	→ (young)		→	–	→ (young) ↑ (old)
	MK801-induced LMA	↓ (0.2)	–	↓ (0.2)	–	↓ (0.3)	–	–	–	↓ (0.2, 0.5)
	Pre-pulse inhibition	↓	→*	→	–	→	–	–	–	→
Cognitive	SWM: T-maze rewarded alternation	–	–	(↓) ^$^	↓	→ (↓ 1s)	–	–	–	→ (↓ 1s)
	SWM: spontaneous alternation; Y-maze/T-maze)	↓** (Y)	→ * (Y)	–	–	–	→ (discrete)	→ (discrete) ↑ (contin.)	–	–
	Spatial novelty-preference	–	–	→ ; ↓***	–	–	–	–	–	→
	Novel-object recognition, short-term	–	–	→ ; ↓***	↓	–	–	–	–	→
	Object displacement, short-term	–	–	–	↓	–	–	–	–	–
	Object displacement, long-term	–	–	–	↓	–	–	–	–	–
	Spatial ref. learning	–	–	→ (+)	→ (Y)	→ (water)	–	–	–	→ (+)
	Cue fear-conditioning	–	–	–	–	↓ (1 shock)	–	–	–	–
	Context fear-conditioning	–	–	–	–	↓ (1 shock)	–	–	–	–
	Reversal learning	–	–	–	–	→ (water)	–	–	–	→ (+)
	Attention (5CSRTT)	–	–	–	–	–	–	–	–	→
	Social memory	(→)*	(→)*	(→)	–	–	–	–	–	→
Negative	Reciprocal sociability	↓*	→*	→	–	–	–	–	–	–
	Non-reciprocal sociability	–	–	→	(→)	–	↓	↓	–	–
	Nest building	↓**	→*	→	–	–	–	↓	–	–
	Anhedonia (sweet preference)	→/↓** (Sacch)	–	→ (Sucr)	–	–	–	–	→ (2% Sucr)	→ (10% Sucr)
	Motivation	–	–	–	–	–	–	–	→ (FST)	–
Anxiety	EPM (young age)	↑**	–	–	–	–	–	–	–	↓
	EPM (medium/old age)	↑	→*	–	–	–	–	–	–	→
	Open field	↑	–	–	–	→	–	→	–	–
	Light/dark-box	–	–	–	–	–	–	–	–	→
	Hyponeophagia	–	–		–	–	–	–	–	↓
m.	Impulsivity (5CSRTT)	–	–	–	–	–	–	–	–	→
	Perseverance (5CSRTT)	–	–	–	–	–	–	–	–	→

Qualifications of results: * phenotype only tested after many days of social isolation/if deficit present, potentially or reportedly dependent on social isolation; ** not present or present in a mild form in group-housed animals but provoked/exacerbated by social isolation; *** phenotype only evoked by decreased environmental enrichment (genotype–environment interaction); () indicative or partially unclear result. ^$^ Deficit very mild, only seen across multiple test (repeated-measures), not within any individual test or protocol. +, plus-maze used for spatial reference testing.

5CSRTT, 5-choice-serial-reaction-time task; contin, continuous; FST, forced swim test of behavioral despair; LMA, locomotor-activity; m., miscellaneous behavioral assessments; PV, parvalbumin (Pvalb); Sacch, saccharine-based sweet preference; Sucr, sucrose-based assessment of sweet preference; Y, Y-maze used for testing of spatial alternation, spatial habituation (novelty-preference), or spatial associative learning; 0.2, 0.5 dose of MK-801 used.

However, there are some technical differences among the published studies using mouse models with NMDAR knockout in PV interneurons (**Table 1**). *Firstly*, they differ partly with respect to the deployed Cre-driver lines. While most recent studies (171, 175, 177–179) used a widely adopted targeted PV-Cre 3’UTR knock-in line (184) that covers the vast majority of PV neurons in neocortex and hippocampus with high specificity (175, 185), the Ppp1r2-Cre line (173) as well as the PV-Cre line (180, 182, 183) used in earlier studies are BAC-transgenic lines that did not target the PV cell population comprehensively. The Ppp1r2-Cre line had originally been reported to express in roughly 70% of the cortical PV cell population, while—conversely—around 75% of the targeted cells were PV-positive (173). A recent re-evaluation in neocortex, however, reported that fewer than 40% of neurons targeted by Ppp1r2-Cre are PV-positive, 10–16% are reelin-positive and 8–12% are somatostatin (SST)-positive (40). In addition, a small fraction of neurons in the hippocampus were also Gad67-negative, and expression in such putative pyramidal cells was reported to expand strongly, especially in CA1, by the ∼20^th^ week of age (173). In general, therefore, the *Ppp1r2*-Cre line cannot be considered a strictly PV-specific driver.

## NMDAR Hypofunction on PV Interneurons and Environmental Stressors Interact to Produce Behavioral Deficits

Another important reason for the potential differences between previous results in different labs, even with the same line, is that NMDAR hypofunction in PV interneurons may interact with environmental risk factors in order to be pathophysiologically harmful. Environmental stressors such as drug abuse, social stress, or even urbanicity can influence both symptoms and treatment success in patients with schizophrenia (189–194). There are now two studies showing that both post-weaning long-term social isolation (181) and a long-term reduction of environmental enrichment starting in early adulthood (176) may provoke or exacerbate various schizophrenia-related deficits in *Grin1^ΔPpp1r2^* mice. A further study demonstrated elevated novelty-induced locomotion in *Grin1^ΔPV^* mice with increasing age (175). Jiang et al. demonstrated that genetic deletion of NMDARs renders PV interneurons more susceptible to oxidative stress which may, in turn, be evoked by environmental stressors such as social isolation (181). It is therefore possible that the susceptibility to stress that NMDAR deletion confers to PV interneurons interacts with differences in housing conditions or other stress-inducing manipulations and thereby causes the differences observed at the behavioral level across studies. Notably, as marked in **Table 1**, the vast majority of testing in the first study in *Grin1^ΔPpp1r2^* mice was conducted in mice that had been socially isolated for at least a week, and sometimes for many weeks, before testing (173). Indeed, for two of these deficits—anhedonia and decreased alternation-based working memory—their dependence on social isolation was subsequently demonstrated explicitly (181, 195). Bygrave et al. demonstrated that even seemingly minor changes in animal housing conditions—as likely exist between different laboratories (reflecting e.g. different regulatory conditions in different parts of the world)—are sufficient to provoke deficits in *Grin1^ΔPpp1r2^* mice, which are otherwise absent in highly enriched conditions (176).

## Oxidative Stress and Zinc Release Constitute Positive Feedback Loops Amplifying NMDAR Hypofunction and Disinhibition

**Figure 4:**
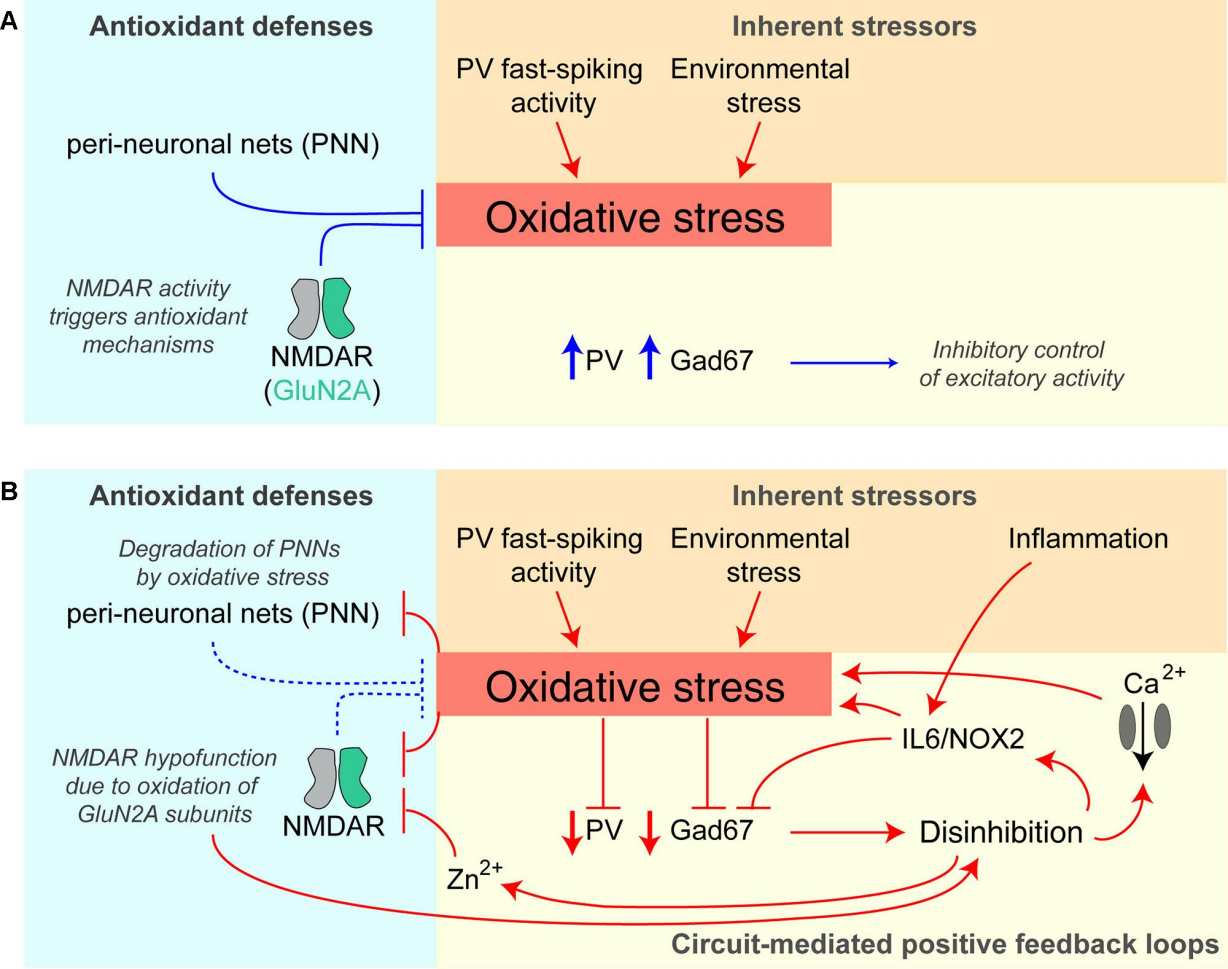
Potential positive feedback loops provoking PV interneuron dysfunction and oxidative stress. **(A)** Healthy baseline state; parvalbumin-positive (PV) interneurons are prone to higher oxidative stress due to their fast-spiking activity and resulting higher metabolism, but perineuronal nets (PNNs) around them and signaling triggered by NMDARs provide antioxidant defenses. They express high levels of PV and Gad67 (producing the inhibitory neurotransmitter GABA) and therefore provide intact inhibition to the neurons in the surrounding circuit. **(B)** Once oxidative stress prevails, e.g. evoked by additional environmental stressors or hypofunction (downregulation/decreased activation) of PV-NMDARs, multiple positive feedback loops are triggered that further amplify oxidative stress and decrease PV interneuron function: the prior protection mechanisms are themselves reduced by oxidative stress—perineuronal nets are degraded by oxidative stress, and specifically, GluN2A-containing NMDARs (that are prominent in PV cells) become oxidized and thereby hypofunctional. Also, the resulting reduction of Gad67 and GABAergic inhibitory output leads to disinhibition of excitatory neurons of the circuit, and *via* IL6/NOX2 and calcium influx through voltage-gated calcium channels increases oxidative stress further. Disinhibition also provokes more glutamate release entailing co-release of zinc ions (Zn^2+^), which block preferentially GluN2A-NMDARs. See (195, 198, 202) for details. Lines with arrows indicate enhancement; lines with vertical line-endings indicate suppression or degradation.

There is evidence that increased oxidative stress in the brain is involved in the pathophysiology of schizophrenia (196, 197) and, due to their high metabolic demands, fast-spiking PV interneurons are thought to be particularly sensitive to oxidative stress (198) (**Figure 4**). In patients with schizophrenia, levels of the antioxidant glutathione (GSH) have been shown to be reduced (199), whereby GSH levels correlated with the severity of negative symptoms of the disease (200). In the rodent *neonatal ventral hippocampus lesion model* of schizophrenia, early administration of *N*-acetylcysteine (NAC), a precursor to GSH, is able to prevent oxidative stress and rescue associated electrophysiological and behavioral abnormalities (201).

Intriguingly, there appear to be *reciprocal* interactions between NMDAR hypofunction and oxidative stress (198) (**Figure 4**). NMDARs are themselves sensitive to oxidative stress because GluN1 and GluN2A*-*NMDAR subunits contain redox-sensitive cysteine residues that can form disulfide bonds resulting in reduced receptor currents (203). Conversely, the activation of NMDARs can promote the transcription of antioxidant genes (204, 205). This coupling of NMDAR activation and regulation of the antioxidant system is thought to equip metabolically active neurons with sufficient antioxidant defenses (198). Therefore, NMDAR hypofunction could reduce such antioxidant mechanisms, and the ensuing oxidative stress then further reduces the conductance of GluN1/GluN2A-containing NMDARs, effectively creating a positive feedback loop. PV interneurons would be particularly vulnerable to this vicious cycle because of their relatively high content of GluN2A-containing NMDARs (117) and their high metabolic activity (206).

A second pathological positive feedback loop involves perineuronal nets (PNNs), which provide an antioxidant defense system, particularly around PV interneurons, but are themselves damaged by oxidative stress. PNNs are reduced in post-mortem tissue from schizophrenia patients, as well as in a variety of related rodent models (206–208).

Beyond cellular mechanisms, NMDAR hypofunction may also induce oxidative stress through circuit-wide effects. Through initiating disinhibition of cortical circuits, NMDAR antagonists can boost levels of the pro-inflammatory cytokine IL-6, with subsequent activation of NADPH oxidase (Nox2) and the production of H_2_O_2_ [reviewed in (209, 210)]. Therefore, repeated or chronic global NMDAR hypofunction—as induced by multiple applications of ketamine, but not by a single injection—leads to an IL-6/Nox2–mediated reduction of GABAergic inhibition from PV interneurons specifically (202, 211). Complementing this finding, interneuron-specific deletion of NMDARs increases reactive oxygen species (ROS), albeit in both inhibitory and excitatory cortical neurons (181). Notably, IL-6 is also increased by immune challenges (212) which in this way—and particularly during development—may contribute to disinhibition independently from (but synergistically with) an NMDAR-dependent mechanism (**Figure 4**).

Environmental stressors, in turn, may lead to oxidative stress if NMDARs on PV interneurons are hypofunctional, thereby provoking damage to cortical circuits that lead to schizophrenia-related deficits. Interestingly, chronic treatment with the antioxidant apocynin was reported to improve many social isolation–induced behavioral deficits in *Grin1^ΔPpp1r2^* mice, including nest building, spontaneous alternation, and pre-pulse inhibition, while also normalizing the production of ROS (181).

A similar circuit-level pathological positive feedback loop may revolve around zinc: zinc is co-released with glutamate by glutamatergic synapses and reduces the open probability (at low concentrations) and blocks (at high concentrations) preferentially GluN2A-containing NMDARs (213–215). In this way, disinhibition in the circuit leads to more zinc release, causing GluN2A-NMDAR hypofunction in PV interneurons, and thereby more disinhibition (**Figure 4**).

## NMDAR Hypofunction in PV Cells as a Risk Factor for Schizophrenia

A model that emerges from this work is that NMDAR hypofunction in PV interneurons is likely to be a *risk factor* for developing schizophrenia, but may not be sufficient on its own. Other (risk) factors, such as environmental stress (potentially causing oxidative stress at the cellular level), or acutely occurring NMDAR hypofunction in other cell types of the circuit [see below and (175)] need to be present and to interact with the cellular consequences of reduced NMDAR expression in PV interneurons to provoke symptoms of schizophrenia (**Figure 4**). Moreover, it is interesting to note that NMDAR knockout in PV interneurons provokes molecular and electrophysiological endophenotypes of schizophrenia potentially *predisposing* the circuit to future insults. For example, *Grin1^ΔPpp1r2^* mice show reduced expression of GAD67 (as also seen in patients with schizophrenia (173) and excess amphetamine-induced accumbal dopamine release aside blunted prefrontal dopamine release (40). The same dopaminergic phenotype was seen in *Grin1^ΔPV^* mice (40).

## Does NMDAR Hypofunction in PV Interneurons Induce Schizophrenia Only When Occurring During Development?

Against this backdrop of discrepancies between studies (**Table 1**), it has also been proposed that NMDAR hypofunction in PV cells may cause schizophrenia-related deficits only if it occurs at a young age but not if it begins after adolescence (173). For example, NMDAR blockade by ketamine leads to reduced PV expression (as also seen in neocortical and hippocampal post-mortem tissue from schizophrenia patients) if the drug is administered during adolescence but not in adulthood (126). It has also been reported that juvenile NMDAR ablation in PV interneurons in mice leads to schizophrenia-related deficits, while ablation later in life does not (78, 216). According to this view, the P7-onset of the Ppp1r2-promoter activation, potentially in combination with the 2.1-kB-short distance between lox-P sites used in the Belforte et al. study (173), which reported a wide range of deficits, might result in NMDAR hypofunction in a critical juvenile developmental window, while the ∼P14-onset of the PV-Cre promoter (174), potentially in combination with a slightly (3.3 kB) (175) or even a much larger distance between loxP sites (12 kB) (177, 178) achieves significant NMDAR hypofunction only in adulthood and is therefore less effective in causing deficits (**Table 1**).

Indeed, some evidence aligns directly with this view. Most importantly, two studies from the Nakazawa lab have shown directly that NMDAR ablation which is stochastically delayed by using the 12-kB-long inter-loxP distance responder line does not lead to a schizophrenia-related phenotype even when combined with long-term social isolation (see **Table 1**), while ablation of GluN1 at an earlier time point using the 2.1 kB inter-loxP distance responder line *does* (40, 173).

However, when looking across the large body of studies as a whole (**Table 1**), the argument is not always empirically supported. For example, when using the same early-onset Ppp1r2-Cre driver line and only a slightly larger distance between loxP sites, we (176) obtained a rather different phenotype from the originally published one (173).

## Acute Global NMDAR Hypofunction in Mice With Chronic PV-Specific NMDAR Hypofunction as a Model of Catatonic Schizophrenia

A single exposure to the NMDAR blocker MK-801 reduces NMDAR expression in PV interneurons but increases expression of NMDARs in all neocortical neurons combined (i.e. presumably in excitatory cells, **Figure 2**) (117). This means that a brain that has previously been exposed to an NMDAR blocker resembles a mouse line with NMDAR hypofunction in PV cells. In such a brain, the balance of NMDAR-mediated contribution to excitation is shifted between the excitatory and PV inhibitory populations of neurons, and when a NMDAR blocker is applied to such a brain—i.e. when *global* NMDAR hypofunction occurs—the consequences are expected to be different. In line with these considerations, we found that MK-801 may induce cognitive and negative deficits of schizophrenia in *Grin1^ΔPV^* mice at concentrations lower than those required in wild type animals to produce similar effects (175). Again, this argues that NMDAR hypofunction in PV interneurons is a *risk factor*—rather than a direct cause—of schizophrenia.

However, the most pronounced phenotype observed with MK-801 application at moderate doses in *Grin1^ΔPV^* mice was the induction of catalepsy, with mice often remaining in the same posture for several minutes. Such cataleptic episodes alternated abruptly with phases of increased locomotion and various forms of stereotypic movement, including circling and repetitive head-shaking (175). Those symptoms are most akin to *catatonia*—a condition that can occur in, but is not specific for, schizophrenia. Catatonic episodes can last for days or even weeks and can be resistant to antipsychotic drugs (217). We also observed pronounced prefrontal delta-frequency (4 Hz) oscillations induced by MK-801 application in these mice (175). An abnormal enhancement of delta oscillations in the awake state has also been described in patients with schizophrenia (85), and it would be intriguing to investigate their presence in patients with catatonic schizophrenia, specifically. These considerations suggest that PV-specific NMDAR hypofunction may be a necessary pre-condition particularly for catatonic schizophrenia patients. This predicts that repeated episodes of pronounced NMDAR antagonism may produce first the *pre-condition* (downregulation of NMDARs specifically in PV cells) and subsequently the *trigger* (global NMDAR hypofunction) of catatonic episodes.

## The Role of NMDAR Hypofunction in Cortical Excitatory Cells

Most studies relevant to the potential consequences of NMDAR hypofunction in *excitatory* cortical cells have not been designed to study the relevance to schizophrenia, but rather to investigate the role of NMDAR-dependent synaptic plasticity in various forms of learning and memory. Despite early demonstrations that associative spatial learning in the Morris water maze (MWM)—but not non-spatial, visual discrimination learning—was impaired by pharmacological blockade of NMDARs (218), it was subsequently shown that rats treated with NMDAR antagonists were in fact perfectly capable of acquiring the MWM task if they were given drug-free pre-training prior to subsequent spatial testing (219–221).

Likewise, the evidence from genetically modified mouse studies that NMDARs on hippocampal principal cells are required for associative memory formation is equivocal at best. Initially, genetically targeted NMDAR ablation (**Table 2**) focused on subsets of cell types such as the principal cells of CA1 (188) and CA3 (222, 223). It was reported that ablation of NMDARs specifically from the CA1 hippocampal subfield impaired associative spatial memory in the water maze (188). However, the first Cre-Driver line (T29-1, CamKIIα-Cre) used in this study to target CA1 excitatory cells was later found to express across the vast majority of excitatory cells of the hippocampus, neocortex, and peri- and enthorhinal cortex (224, 225). It was subsequently demonstrated that NMDARs in CA1 and/or dentate gyrus are necessary for making correct choices related to *ambiguous* cues associated with overlapping or competing memories, rather than mediating associative spatial memory formation or recall as such (226). NMDARs in CA3 are likewise not essential for associative spatial memory formation, but have been claimed to support memory recall with incomplete cues as well as performance on a delayed match-to-place (serial reversal) memory task (222, 223).

**Table 2:** Deficits induced by putative genetic NMDAR ablation in excitatory cells of the cortex, including hippocampus. Results of behavioral tests conducted in the indicated studies (top row) measuring rodent correlates of schizophrenia in the positive, cognitive, and negative domain as well as anxiety (see left two columns) in distinct double-transgenic conditional knockout lines (stated in rows 2–4). Green →, no change; magenta, schizophrenia-related deficit; orange, deficit seen in specific test phases or with specific test conditions but not others; blue, apparent improvement of function or opposite of the expected. ↑, increase of behavioral measure; ↓, decrease of behavioral measure. *Studies*: Tsien 1996 (188), McHugh 1996 (227), Tatard-Leitman 2015 (228), Nakazawa 2002, 2003 (222, 223), Finlay 2015 (229), Bannerman 2012 (226), Rompala 2013 (230), Vieira 2015 (231), Brigman 2010, 2013 (224, 232). *Cre-Driver lines*: T29-1/CamK-Cre (225) [originally assumed to target CA1 pyramidal cells, but later shown to target excitatory cells across neocortex and hippocampus, e.g. (224); Tg*^Cre4^*/CamK-Cre (233); KA1/G32-4-Cre expresses in CA3 pyramidal cells (222); *Tg*CN12*;Tg*LC1 expresses in excitatory cells of CA1 and dentate gyrus (226); G35-3-Cre expresses in excitatory neurons of the hippocampus and the frontal, parahippocampal, and sensory cortex, esp. in layers 2/3 (230). AAV-CamK, Cre is expressed from a locally injected AAV-vector and driven by the CamKIIα-promoter (229). *Floxed-Grin1 and -Grin2B responder lines*: Tonegawa (Jax# 005246) (188), Seeburg (186), Holmes (224), Monyer (234).

	Publication	Tsien 1996 McHugh 1996	Tatard-Leitman 2015*	Nakazawa 2002, 2003; Finlay 2015	Bannerman 2012	Rompala 2013	Finlay 2015; Vieira 2015	Brigman 2010, 2013 v. Engelhardt 2008

Line	Driver	CamK: T29-1	CamK: T29-1	KA1/G32-4; AAV-CamK	*TgCN12;TgLC1*	KA-1/G35-3	AAV-CamK	CamK: T29-1; Tg*^Cre4^*
	Responder	Tonegawa	Tonegawa	Tonegawa	Seeburg	Tonegawa	Tonegawa	Holmes; Monyer
	Gene, loxP–loxP distance	Grin1, 12 kb	Grin1, 12 kb	Grin1, 12 kb	Grin1, 3.3 kb	Grin1, 12 kb	Grin1, 12 kb	Grin2B, 1 kb; 2 kb
	Targeted region	CA1 (& cortex)	Cortex/HC	CA3	CA1, DG	Cortex, HC	mPFC	Cortex, HC
Positive	Novelty-induced LMA	–	↑	–	↑	→	–	–
	MK801-induced LMA	–	–	–	–	→	–	–
	Amphetamine –induced LMA	–	–	–	–	→	–	–
	Pre-pulse inhibition	–	–	–	–	↓	–	–
Cognitive	SWM: spontaneous alternation	–	↓ (T) ^&#^	–	–	→ (Y)	–	↓ (T, Y) ^#^
	Spatial novelty-preference	–	–	–	–	→ (Y, 3h delay)	–	–
	Novel-object recognition, short-term	–	–	–	–	→, ↓ ^£^	–	↓
	Spatial reference learning	↓ (MWM)	–	→ (MWM)	↓ (MWM^&^, RM, Y)	–	–	↓ (MWM)
	Spatial ref. learning w. partial cue	–	–	↓	–	–	–	–
	Spatial reference learning—beacon	–	–	–	↓ MWM) ^&^	–	–	–
	Spatial reversal learning	–	–	–	↓ (MWM)	–	–	↓
	Visual discrimination assoc. learning	→ (MWM)	–	–	→ (MWM, T)	–	–	→ (T.Sc.), ↓ (T)
	Visual discrimination reversal	–	–	–	→ (MWM, T)	–	–	↓ (T.Sc.)
	Context/cue fear-conditioning (FC)	–	–	–	–	→ (context FC)	→ (cue FC)	→ (delay); ↓ (trace)
	Cue-discrimination FC	–	–	–	–	–	↓	–
	Fear-memory extinction	–	–	–	–	–	↓	–
	Operant learning	–	–	–	–	–	–	→
	Social memory	–	–	→	–	(→)	↑	–
	Sustained attention, 5CSSRT	–	–	→	–	–	→	–
	Inattentiveness, 5CSSRT	–	–	→	–	–	→	–
	Response latency, 5CSSRT	–	–	→	–	–	→	–
								
Negative	Reciprocal sociability	–	–	–	–	→	→	–
	Non-reciprocal sociability	–	↓	↓	–	–	–	–
	Nest building	–	↓	–	–	–	–	–
	Anhedonia (sweet preference)	–	–	–	–	→ (Sacch.)	–	–
	Motivation (reward latency)	–	–	–	–	–	–	→
misc.	Open field: anxiety	–	–	–	–	→	–	–
	Motor impulsivity, 5CSSRT	–	–	↑	–	–	→	–
	Perseveration, 5CSSRT	–	–	↑	–	–	→	–
	Electrophysiology	Larger & less specif. CA1 place fields	↓ evoked power ↑ baseline power	Smaller/absent CA1 place fields w. partial cue				↓ cellular LTP

Qualifications of results: () indicative result, not properly measured or nor clear result. ^$^ Deficit very mild, only seen across multiple test (repeated-measures), not within any individual test or protocol. ^&^ A deficit occurs in spatial reference memory in the MWM if the starting point is further away from the correct goal (platform) than from the incorrect goal (distracting beacon in beacon-version or opposing quadrant in standard version), there are also deficits in the Y-maze and radial-maze; i.e. deficits are seen in all cases were multiple and partially undefined spatial cues are used, rendering the reference-frame ambiguous. ^#^ Note that this result may be confounded by more fundamental aberrations in spatial processing. ^£^ Impairment only seen with longer delays or large memory load (five objects instead of two). * This mouse line also shows increased baseline power and decreased evoked power in the theta, beta, and gamma range, and also reduced expression of CCK (in neocortex), 5HT-2A (in hippocampus), dopamine D2 receptor (D2R) (in neocortex), and GRIK2 (both), while cortical expression of GAD67, PV, somatostatin (SST), D1R, GluA1/2/3/4, and 5HT-1A/2B/2C is unchanged (228).

5CSRTT, 5-choice-serial-reaction-time task; assoc., associative; FC, fear-conditioning; LMA, locomotor-activity; MWM, Morris water maze, ref., reference; RM, radial-arm maze; Sacch., saccharine-based assessment of sweet preference; T, T-maze; Y, Y-maze; T.Sc. touch-screen–based operant assay used for testing.

A later re-assessment of a mouse line with NMDAR ablation in cortical excitatory cells (*Grin1^ΔCamKIIα^* mice) across a wide range of schizophrenia-related behaviors and endophenotypes demonstrated that this line shows impairments across the major symptom domains related to schizophrenia (see **Table 2**), including robust deficits in alternation-based spatial working memory, nest building, social interaction, and novelty-induced hyperlocomotion (228). *Grin1^ΔCamKIIα^* mice also displayed electrophysiological endophenotypes relevant to schizophrenia (235–237), including an elevated power of baseline gamma, theta, and beta oscillations and decrease of stimulus-evoked oscillations in these frequency bands (228). Interestingly, pyramidal cells were also more excitable in response to current injections. In contrast to the phenotype induced by pharmacological or PV-specific NMDAR hypofunction, in mice with NMDAR hypofunction in *excitatory* cells the expression of PV and Gad67 was not altered in the neocortex or hippocampus (228). This demonstrates that several behavioral and electrophysiological endophenotypes seen in schizophrenia could be caused by NMDAR hypofunction in excitatory pyramidal cells *alone* and do not require reduced Gad67 expression in PV cells. Notably, the level of cortical Gad67 expression also varies strongly among patients with schizophrenia (148), suggesting that its reduction might not be a necessary element of disease development.

In a similar approach, following ablation of GluN2B from excitatory cells across the forebrain (*Grin2B^ΔCamKIIα^* mice), a broad range of profound memory deficits were found, including chance-level performance in novel-object recognition and alternation-based spatial working memory, but also in spatial long-term memory tasks, and in visual discrimination learning (234). In contrast, when GluN2B-ablation was restricted to CA1 and dentate gyrus excitatory cells, basic spatial associative learning was left intact, although deficits in spatial reversal learning and (to a lesser extent) alternation-based working memory remained (234).

However, there are two caveats to these observations: firstly, on the one hand, some deficits seen after ablation of *Grin1* or *Grin2B* in excitatory cells (**Table 2**) may be difficult to reconcile with the symptom-profile of schizophrenia, including a wide range of deficits in basic long-term associative spatial memory tasks, fear extinction and cue-discrimination in fear-conditioning, as well as enhanced social memory (188, 226, 229, 231). Note, however, that these learning abnormalities could reflect abnormal credit assignment with *inappropriate* associations between cues resulting from aberrant assignment of salience, which is suspected to be a core deficit in schizophrenia (238–240).

Secondly, a study using a different Cre line that expresses in a wide range of cortical excitatory cells (although lacking expression in deeper layers in some areas of neocortex and in all layers of some parts of neocortex, e.g. retrosplenial cortex, and in a considerable proportion of hippocampal pyramidal cells), found almost no schizophrenia-related deficits (230). Therefore, it is probably a rather specific subset of excitatory cells, which likely include—but are not limited to—hippocampal pyramidal neurons, in which NMDAR hypofunction may lead to schizophrenia-related deficits.

## The Role Of NMDAR Hypofunction In Thalamic Neurons And The Importance Of GluN2C

A third potential location of NMDAR hypofunction promoting schizophrenia pathogenesis is the thalamus—especially its reticular nucleus (RTN), whose neurons are GABAergic, PV-positive, and rely largely on GluN2C-containing NMDARs for their excitatory drive (78, 114). GluN2C-NMDARs are also expressed in the glutamatergic cells of the relay and unspecific nuclei of the thalamus (241).

Work by John Lisman’s group supports a model in which blockade of GluN2C-containing NMDARs in both the excitatory relay cells and the inhibitory PV-positive RTN cells of the thalamus might be the key mechanism by which NMDAR antagonists like ketamine produce increased delta oscillations. Ketamine—at a psychotomimetic dose in humans—strongly blocks GluN2C-containing receptors, but affects GluN2A/2B-containing NMDARs to a lesser degree, since the former are about three times more sensitive to this drug compared to the latter (114). Additionally, the channel is relatively insensitive to Mg^2+^ ions, allowing it to provide a tonic excitatory drive at the resting potential of thalamic neurons (78, 114, 107). GluN2C-NMDAR blockade by ketamine would be expected to hyperpolarize both cell types, thereby activating T-type calcium channels Cav3.3 (slow) in PV-positive RTN cells and Cav3.1 channels (fast) in glutamatergic relay cells (242, 243), inducing endogenous depolarization-cycles that translate into thalamocortical delta oscillations. This hypothesis is supported by the finding that prefrontal delta oscillations are induced by ketamine and are (abnormally) present in the wake state of patients with schizophrenia (85, 114).

We also observed strong prefrontal delta oscillations after application of MK-801 in *Grin1^ΔPV^* mice (175). A combination of reduced activation of inhibitory, PV-positive RTN cells due to NMDAR ablation in *Grin1^ΔPV^* mice on one hand, and an additional blockade of remaining NMDARs in thalamic relay cells by MK-801 on the other, could lead to hyperpolarization and activation of Cav3.1 channels in relay cells, and hence the induction of delta oscillations [as observed in (175)]. Also note that Cav3.3 is a schizophrenia risk gene, and the risk-associated mutation renders this channel hypofunctional (244), thereby potentially producing weaker activation of inhibitory RTN cells. Notably, local infusion of MK-801 specifically into the mediodorsal nucleus of the thalamus (at high doses) and into the RTN (already at low doses) causes increased glutamate release in prefrontal cortex in rodents (118, 119, 121). It should also be noted that specific nuclei in the human (but not the rodent) thalamus also contain PV-positive interneurons and projection neurons, and a degeneration of the latter has been described in post-mortem tissue from schizophrenia patients (245, 246).

## NMDAR Hypofunction in Dopamine Cells, Striatal Neurons, and SST Interneurons

Other candidate cell types which have been implicated in schizophrenia and suggested as a locus of NMDAR hypofunction are SST-positive interneurons (247) and VTA dopamine neurons (78). In contrast to neocortical PV interneurons and pyramidal cells (117), both of these cell types contain GluN2D-NMDARs (78) which—like GluN2C-containing NMDARs—have a higher affinity for ketamine and are largely insensitive to the Mg^2+^-block at resting membrane potential (78); these properties imply that NMDAR antagonists would preferentially reduce excitatory drive in those cells, as opposed to neurons that contain mainly GluN2A/B-NMDARs. *Grin1^ΔSST^* mice still need to be evaluated comprehensively regarding schizophrenia-related deficits, but a first analysis showed that they do not demonstrate the elevated accumbal and blunted prefrontal dopamine release after amphetamine application and elevated amphetamine-induced locomotion seen in *Grin1^ΔPpp1r2^* and *Grin1^ΔPV^* mice (40).

A further locus of NMDAR hypofunction might be VTA dopamine neurons. These cells are a putative hub of schizophrenia pathology and feature a surprising decrease in expression of NMDARs in a prominent schizophrenia mouse model with striatal D2R overexpression (248). Selective ablation of NMDARs from dopamine neurons does not induce core endophenotypes of schizophrenia such as deficits of working memory, short-term object memory, sociability, or pre-pulse inhibition, nor elevated novelty- or amphetamine-induced locomotor activity (249, 250). However, NMDAR knockout from dopamine neurons causes inappropriate generalization in fear-learning across cues as well as impaired visual discrimination and spatial reference learning (250, 251).

Finally, NMDAR ablation has been experimentally confined to medium spiny neurons (MSNs) of the basal ganglia using the RGS9-Cre driver line. Mice lacking all NMDARs (i.e. *Grin1*) in MSNs display deficits in motor learning [on the rotarod (187)], while mice lacking GluN2B-containing NMDARs in MSNs have difficulties with visual discrimination learning (i.e. long-term associative memory) and its reversal, while simple operant learning is intact (232). It is unclear to what extent these deficits are relevant to schizophrenia, and the overall behavioral profile suggests that striatal MSNs are not a major site of NMDAR hypofunction in the pathogenesis of this disease.

Deficits of accurate discrimination between sensory stimuli and resulting over-generalization during learning—seen with NMDAR ablation in the VTA and the MSNs—might, however, point to aberrations of learning processes that could contribute to aberrant salience (239) and the formation of inappropriate associations and delusions in humans (252).

## Conclusions

In summary, NMDAR hypofunction in multiple *specific* neuron types may contribute to the causation of symptoms in schizophrenia. Indeed, given that genetically driven schizophrenia will potentially produce NMDAR hypofunction across all cells, it may be unrealistic to think that deficits in any one cell type may explain the disorder. It is possible that *acute* blockade of NMDARs by NMDAR antagonists such as ketamine causes schizophrenia-related symptoms by preferentially reducing the activity of neurons that express NMDARs containing GluN2D (SST interneurons, dopamine neurons) and GluN2C (thalamic relay cells, PV-positive cells of the thalamic RTN and cortex, and presynaptic terminals of PV interneurons). NMDAR hypofunction in the *chronic* disease state, in contrast, is probably localized somewhat differently, and GluN2A/B-containing NMDARs likely play a role as well. While there is little compelling evidence that NMDAR hypofunction in PV-positive interneurons causes schizophrenia *directly*, it is likely a key *risk factor* for this disease. It renders PV interneurons more prone to oxidative stress which may be exacerbated by inflammation, environmental stress, or interactions with other risk genes.

The consequences of NMDAR hypofunction in other interneurons, such as SST and CCK cells still remain to be comprehensively assessed. NMDAR ablation in dopamine or striatal MSNs, in contrast, does not seem to induce a schizophrenia-related spectrum of deficits, although the observed specific aberrations in valuation and over-generalization during learning might contribute to positive symptoms. Nevertheless, some of the symptoms of schizophrenia—especially in the cognitive domain—may be caused by NMDAR hypofunction in the thalamus and in specific subsets of cortical pyramidal cells. We speculate that treatments that increase NMDAR function, reduce oxidative stress, or directly boost the function of PV and other interneurons, could conceivably be beneficial in these cases, especially if applied in the prodrome or early in schizophrenia (253). Given the limited success of previous approaches to enhance NMDAR-function rather directly, it is likely that any future success will depend on a better understanding of the interacting role(s) of NMDARs in different cell types within multiple different neural circuits.

## Author Contributions

AB, KK, and DK conducted literature research and prepared the manuscript, which was revised by all authors.

## Funding

The research that was conducted by the authors and is discussed in this review was funded by a Sir Henry Wellcome Postdoctoral Fellowship (DK, DMK; grant number 098896), a predoctoral OXION fellowship from the Wellcome Trust (AMB), a predoctoral LGFG fellowship from the state of Baden-Württemberg (KK), the John Fell Fund of the Oxford University Press (DK, DMB), the DFG (DK), grants from the Medical Research Council (DMK, DMB), and an Investigator Award from the Wellcome Trust (DMK). The publication of this article is funded by a grant from the Deutsche Forschungsgemeinschaft (DFG, KA/4594/2-1) and Ulm University.

## Conflict of Interest

The authors declare that the research was conducted in the absence of any commercial or financial relationships that could be construed as a potential conflict of interest.
